# Randomised clinical trial investigating memory training for recovery-adolescents in addressing psychiatric concerns among adolescents in Iraq

**DOI:** 10.7189/jogh.15.04111

**Published:** 2025-05-05

**Authors:** Laura Jobson, Hussain Malallah, Sayed Jafar Ahmadi, Daniel McAvoy, Arul Earnest, Kelsey Vaughan, Latef S Berzenji, Shkofa Mohammad, Azi Berzengi

**Affiliations:** 1 Turner Institute for Brain and Mental Health and School of Psychological Sciences, Monash University, Melbourne, Victoria, Australia; 2 National Institute for Human Rights, Kirkuk, Iraq; 3 Psychology Faculty, Bard College, Annandale-On-Hudson, New York, USA; 4 Centre for Humanitarian Leadership, Deakin University, Melbourne, Victoria, Australia; 5 Biostatistics Unit, School of Public Health and Preventive Medicine, Monash University, Melbourne, Victoria, Australia; 6 Bang for Buck Consulting, Amsterdam, the Netherlands; 7 Al-Qalam University College, Kirkuk, Iraq; 8 Kirkuk Cultural and Social Association, Kirkuk, Iraq; 9 Department of Clinical Psychology and Psychological Therapies, University of East Anglia, Norwich, UK

## Abstract

**Background:**

In this randomised clinical trial, we investigated the efficacy of MEmory Training for Recovery-Adolescent (METRA) in improving psychiatric symptoms among adolescents in Iraq.

**Methods:**

In the study, we included adolescents aged 10–19 years with heightened psychiatric distress living in Kirkuk. It was a parallel-group trial comparing METRA with treatment as usual (TAU), with a three-month follow-up. The study occurred between July 2023 and January 2024. Participants assigned to METRA received a 10-session group-intervention comprised of memory specificity training and writing for recovery. Assessments occurred at baseline, post-intervention, and three months after treatment. Primary outcome measures were self-reported posttraumatic stress disorder (PTSD) and depression symptoms post-intervention. Secondary outcomes were measures of anxiety and psychiatric difficulties. We also examined the costs and affordability of METRA in a humanitarian context. The sample size for primary analyses included 67 adolescents in the METRA group and 65 adolescents in TAU.

**Results:**

Following the intention-to-treat principle, linear mixed effects models found at post-intervention the METRA group had a 10.96-point decrease (95% confidence interval (CI) = –13.82, –8.09) in PTSD symptoms and a 3.27-point decrease (95% CI = –4.67, –1.87) in depression symptoms. Improvements were maintained at the three-month follow-up. While the time main effects were significant (*P* < 0.001), the group × time interactions were not significant (*P* = 0.61 for PTSD and *P* = 0.71 for depression); thus, there was no evidence that these improvements were superior to the symptom improvements observed in TAU.

**Conclusions:**

In this study, we found that while METRA was not more effective than TAU, it was less costly, offering an option for replacing current practice. The findings highlight a need for further research in this area of global mental health.

**Registration:**

Australian New Zealand Clinical Trials Registry (ACTRN12622001413718).

There are more adolescents in need of humanitarian aid and protection than at any other point since World War II [1]. Humanitarian crises disproportionately impact adolescents [2], including increasing the risk of posttraumatic stress disorder (PTSD), depression and anxiety [3-5]. Despite adolescents facing these mental health challenges, many do not receive evidence-based interventions, with the mental health treatment gap being as high as 85% in low- and middle-income countries (LMICs), which represents an urgent global health priority [6,7]. Over the past decade, there has been increased attention on mental health treatments among adolescents in LMICs [7,8], with studies showing the effectiveness of a range of approaches, including cognitive behaviour therapy, school-based programmes, narrative exposure therapy and psychosocial assistance [8,9]. Yet, little research has examined the development and evaluation of evidence-based mental health interventions for adolescents or considered their affordability in humanitarian contexts [10]. Additionally, very few studies have examined mental health interventions for adolescents in Iraq, despite it being stressed that interventions examined in one humanitarian context may not be adequate for another context [8,9]. Therefore, in this study, we examined whether MEmory Training for Recovery-Adolescent (METRA) improved mental health among adolescents in Kirkuk, northern Iraq.

Iraq continues to face protracted humanitarian concerns [11]. Over the past 20 years, the country has faced war, conflict, violence, insecurity, political instability, lack of education and medical facilities, and challenges meeting basic human needs [12–14]. Millions of Iraqis have been, or continue to be, displaced, resulting in adolescents facing protection and security risks and difficulties accessing education and health care [12–14]. Kirkuk is a disputed region [15]. The population is composed of various ethnic (*e.g.* Arabs, Kurds, Turkmens) and religious (Sunni and Shia Muslims, Christians) groups [15]. Since 2003, Kirkuk has experienced severe human rights violations, faced considerable conflict, and been affected by militant groups, which have influenced employment and education, community well-being, living and health standards, and cultural activities [15]. Thus, adolescents in this region have been exposed to conflict and human rights violations for most of their lives. While these factors are specific to Kirkuk and the broader Iraqi context, many humanitarian settings share similar challenges – the effects of long-term conflict, socio-political instability, and cultural diversity. In recent years, Iraq has experienced relatively greater stability, and there has been a call for the global community to prioritise mental health [12,13]. Nevertheless, it remains difficult to address mental health in Iraq [12,13]. It is estimated that around 20% of Iraqis have a mental health condition, and this number is increasing [12]. The few studies focusing on Iraqi adolescents also indicate increased rates of mental health concerns, including PTSD and depression [14,16].

While Iraq has had a stand-alone mental health policy since 2017, it does not have specific strategies for adolescent mental health [13]. Moreover, Iraq’s mental health system is not well-developed, as there are limited and disproportionate resources available, given the burden of disease [12,13], an issue faced in many humanitarian settings [17]. Consequently, few adolescents in Kirkuk, and Iraq more broadly, receive evidence-based interventions. Therefore, there is a need for community-based mental health interventions enabling local health care providers to treat adolescent mental health at low cost [8,12,13]. METRA has been designed as a low-intensity intervention that can be delivered in the community, particularly communities affected by humanitarian crises, by community health providers.

There is potential to improve adolescent mental health by utilising low-intensity interventions targeting cognitive difficulties underpinning psychiatric distress [18]. METRA is a group-based intervention for adolescents that focuses on two autobiographical memory disruptions underpinning PTSD and depression. It is comprised of two modules that are delivered over 10 sessions by a facilitator with health training. Module 1 of METRA is based on MEmory Specificity Training (MEST) [19,20] and thus targets difficulties in recalling specific past events. Reduced memory specificity predicts the development and maintenance of PTSD and depression and is associated with psychiatric difficulties that can endure into adulthood [21]. Additionally, reduced memory specificity is associated with cognitive avoidance and rumination, factors known to maintain PTSD and depression [22]. Improving memory specificity can lead to improvements in PTSD and depression [23], including among war-affected youth in Iran [19] and Afghanistan [24]. Module 2 of METRA is writing for recovery and targets the trauma memory through written exposure. Trauma memories are often intrusive, disruptive and distressing [25]. Gold-standard evidence-based interventions for PTSD target the processing of the trauma memory through exposure strategies. These are thought to integrate the trauma memory into the autobiographical memory network and thereby reduce its intrusive, disruptive, and distressing qualities [25]. The focus on trauma exposure approaches has featured in the emerging evidence-based interventions for adolescents with PTSD in LMICs, with a focus on high-intensity psychotherapy [8]. The focus of METRA is to target similar mechanisms (memory specificity, exposure to trauma memories) using a low-intensity approach delivered by community health professionals [6].

METRA has been examined in the humanitarian context of Afghanistan. Afghan adolescent girls offered METRA had fewer symptoms of PTSD, depression, anxiety and psychiatric difficulties at post-intervention than those receiving adolescent health sessions [26]. These improvements were maintained at a three-month follow-up, and adolescents and facilitators reported satisfaction with METRA [26]. Afghan adolescent boys, who were offered METRA following a terrorist attack in Kabul, had significantly greater reductions in symptoms of PTSD, depression, anxiety, and psychiatric difficulties than did those in the control group [27]. These initial findings are promising in suggesting that METRA may be a feasible intervention for adolescents in humanitarian contexts. However, given the unique complexities of each humanitarian context, there is a need to examine METRA in other humanitarian contexts. Thus, we aimed to examine whether we could similarly implement METRA in Northern Iraq.

Additionally, while METRA is noted as being a low-resource intervention, its costs and cost-effectiveness compared to other mental health interventions has not been examined. Across all mental health interventions implemented in humanitarian contexts, there is little known about these topics, despite repeated calls for more evidence in these areas [28–30]. The few available studies use different outcome measures and methods, making them nearly impossible to compare, and none have considered affordability in the context of already low mental health funding in LMICs [31] and particularly constricted resources across humanitarian settings [32–37].

We conducted a randomised clinical trial to investigate the efficacy, cost-effectiveness and feasibility of METRA in addressing psychiatric concerns among adolescents in Iraq. The primary objective was to investigate the efficacy of METRA in improving PTSD and depression symptoms. We hypothesised that METRA would lead to significantly more significant reductions in PTSD and depression symptoms from baseline to post-intervention than treatment as usual (TAU). The secondary objectives were to investigate: 1) the efficacy of METRA in improving general psychiatric symptoms (anxiety, psychiatric difficulties), 2) whether improvements in symptomatology were maintained at three-month follow-up, 3) the mechanisms mediating any treatment effects (*i.e.* whether changes in rumination and avoidance mediated any improvements in PTSD and depression symptomatology), 4) the costs, cost-effectiveness and affordability of METRA in a humanitarian context, and 5) the feasibility and appropriateness of METRA.

## METHODS

### Design

Monash University Human Research Ethics Committee approved the study (approval number 35071). It was a randomised controlled trial of the preregistered trial (ACTRN12622001413718). We used a parallel trial design to compare METRA to TAU and assessed participants at baseline, post-intervention, and three-month follow-up.

### Participants

**Figure 1:**
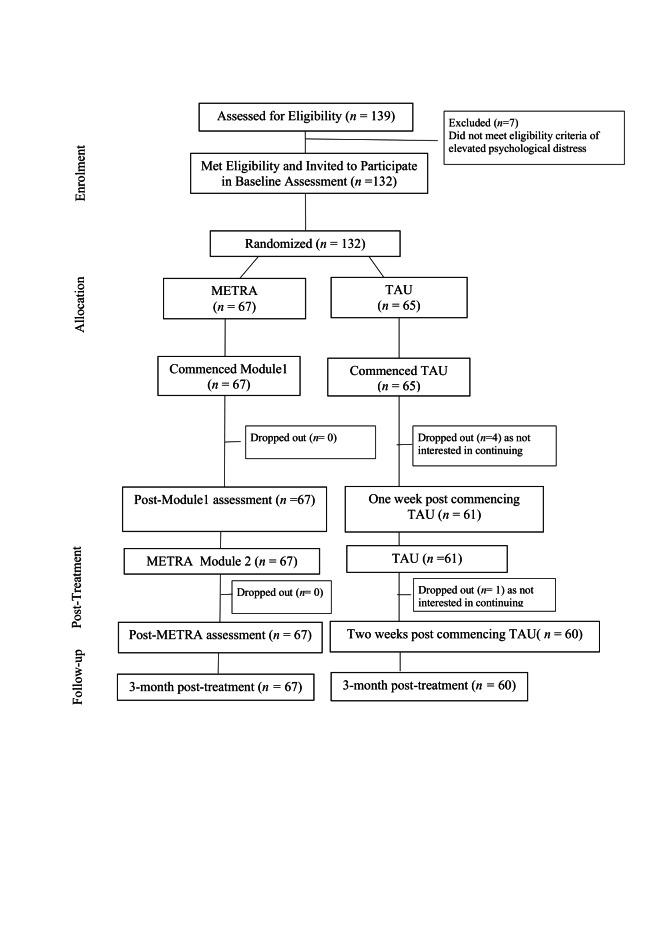
Flowchart of participant recruitment and assessment.

We approached adolescents through a community organisation in Kirkuk, Iraq. We screened 139 adolescents with a mean age (x̄) = 13.12 years (standard deviation (SD) = 1.94). Of this number, 132 participants aged 10–19 years (x̄ = 13.17; SD = 1.95) met eligibility criteria, and we invited them to participate and randomly allocated to METRA (n = 67) or TAU (n = 65). Eligibility criteria were: 1) aged 10–19 years, 2) experiencing elevated posttraumatic distress, defined as ≥25 on the Child Revised Impact of Event Scale-13 [38] and/or >12 on the Mood and Feeling Questionnaire [39], and 3) able to complete the tasks in Arabic. A priori power analysis was undertaken using G*Power, version 3.1.9.7 (Heinrich Heine University Düsseldorf, Düsseldorf, Germany) (repeated measures analysis of variance) for PTSD/depression outcomes. We calculated that a sample size of 62 participants per arm would provide adequate power (80%) at a 5% significance level, assuming a moderate effect size of 0.15. As we had no prior information on the within-subject correlation, we opted to choose a medium level of correlation (ρ = 0.5). CONSORT flow diagram is presented in **Figure 1**, and the checklist in Table S1 in the **Online Supplementary Document**.

### Procedure, randomisation, and blinding

Enrollment began in July 2023, and data collection ended in January 2024. We collected informed written consent from adolescents and parents/guardians. Following baseline assessment, participants were individually randomised in a 1:1 ratio to METRA or TAU using block randomisation (sizes two and four) generated by the trial statistician (blinded). We used consecutively numbered, sealed, opaque envelopes to conceal the allocation. A researcher in Kirkuk (independent of intervention delivery and assessment administration) monitored the generation of the allocation sequence, participant enrolment, and assigning participants to interventions. Assessments were conducted in Arabic by independent assessors who had no therapeutic relationship with participants and were blind to group allocation, and randomisation information was not accessible to these researchers. It was not possible to blind the therapists or the participants given the interventions under investigation. However, the outcome assessors were blind to intervention allocation and the therapists and participants were instructed not to discuss their allocation with the assessor. This was also stressed at the start of the outcome assessment tasks to minimise any potential bias the assessor may have when conducting the outcome assessments. We also requested that participants refrain from discussing the details of the study or the components of the interventions with others.

### Measures

#### Children Revised Impact of Event Scale 13 (CRIES-13)

The CRIES-13 is a 13-item measure of PTSD [38]. Participants responded to items on six-point Likert scales (zero indicating ‘not at all’ and five indicating ‘often’). Total scores were added for each participant as an index of PTSD symptomatology, and total scores ranged from zero to 65, with higher scores indicating more significant PTSD symptoms [38]. The Arabic version of the CRIES-13 has good psychometric properties [40] and is routinely used with Middle Eastern populations, including Iraqi adolescents [9,40,41]. In this study, Iraqi psychologists and social workers reviewed the items and deemed no changes were required to the Arabic version of the CRIES-13. We found internal consistency to be good (Cronbach α = 0.73).

#### Mood and Feelings Questionnaire - short form (MFQ-SF)

The MFQ-SF contains 13 items assessing depression [39]. Adolescents rated items as not true (zero points), sometimes true (one point), and true (two points). Items were summed to provide a total depression score, with higher scores indicating greater depression severity [39]. We used the Arabic version of the MFQ, which has good psychometric properties [42]. Iraqi psychologists and social workers reviewed the items and deemed no changes were required to the Arabic version of the MFQ-SF for use in this study. Internal consistency was found to be good (Cronbach α = 0.87).

#### Revised Children’s Manifest Anxiety Scale (RCMAS)

The RCMAS is a 37-item questionnaire assessing anxiety [43]. Scores on the anxiety items were totalled to give a total anxiety score, with higher scores indicating worse anxiety. The RCMAS has good psychometric properties [43] and has been used with Iraqi youth [44]. In this study, internal consistency was good (Cronbach α = 0.76).

#### Strengths and Difficulties Questionnaire (SDQ)

The SDQ is a 25-item self-report questionnaire that screens adolescents for psychiatric difficulties and prosocial behaviour over the past six months [45]. Given the focus of the study, we used the total difficulties as an index of psychiatric difficulties. The SDQ has been used with Iraqi youth [46]. Internal consistency was adequate (Cronbach α = 0.65).

#### Children’s Response Styles Questionnaire (CRSQ)

The CRSQ assesses ruminative, distractive, and problem-solving responses [47]. Given the focus on rumination as a proposed change mechanism, we only used the rumination subscale. The rumination subscale included 13 items, with participants responding to items on four-point Likert scales (zero referring to ‘almost never’ and three referring to ‘almost always’), with scores ranging from zero to 39 and higher scores indicating a greater tendency to engage in rumination [47]. In the current study, internal consistency was good (Cronbach α = 0.73).

#### Cognitive Avoidance Questionnaire (CAQ)

The CAQ is a 25-item measure of the tendency to employ cognitive avoidance strategies when dealing with threatening intrusive thoughts [48]. Participants responded to statements on five-point Likert scales (one indicating ‘not at all typical’ and five indicating ‘completely typical’). For each participant, we calculated an overall total score, with higher scores indicating more significant avoidance. In this study, internal consistency was good (Cronbach α = 0.92).

### Interventions

#### METRA

METRA is a manualised group training (eight adolescents/group) comprised of two modules delivered daily over a fortnight, with Module 1 delivered daily in the first week and Module 2 delivered daily in the second week (with a weekend gap between the two modules). METRA was planned to be delivered over 10 weekly sessions [26]. However, in the Afghanistan trials, feedback from the research team and community highlighted security issues, which prevented METRA from being delivered over 10 weeks. Thus, Module 1 was delivered over three mornings and two afternoons within a week and Module 2 was delivered as five daily sessions (with a four-day break between the modules) [26]. In partnership with clinicians and community in Northern Iraq, it was decided that the 10 weekly sessions were also impossible in Iraq (due to feasibility and security concerns). Instead, there was also a preference for fortnightly delivery, with sessions being delivered daily. We adopted this approach as it was essential that implementation in both sites was guided by local teams and the community.

Module 1 is based on MEST [19,20]. Session 1 provided psycho-education developed for METRA that included information about memory, emotional disorders and memory, and memory specificity. This information was delivered verbally by the facilitator and included examples. In sessions one to three, participants recalled specific memories in response to positive, neutral, and negative cues. For homework, participants generated a specific memory for 10 cues. Session four involved exercises using negative and counterpart positive cues, discussions, and exercises to promote metacognitive awareness. Session five included further practice and a summary. Module 2 was a modified form of written exposure therapy and writing for recovery [49–51]. Session one included a brief outline of Module 2. In sessions one to five, adolescents repeatedly wrote about their trauma experience/s for a full 30 minutes. Adolescents were encouraged to write about the details of the trauma(s) as they remember it now (including details of what happened, thoughts and feelings, worst aspects of the event, how the event had touched their life). After 30 minutes, the facilitator asked the adolescents to finish up and ensured that they were ready to leave. Participants left their writing books behind between sessions and the facilitator read the narratives to ensure participants had understood the task and were engaging appropriately. All sessions were 60 minutes.

#### TAU

Following the baseline assessment, the TAU group received individual trauma-informed interventions that a trained social worker and psychologist delivered from a local non-government organisation (NGO). The interventions offered were those that would have been routinely offered to adolescents with psychological stress and were devised based on the adolescent’s need and severity of symptoms. TAU intervention included trauma-focused talking therapy, meditation, art therapy, and psychological health interventions (referrals to the Psychological Health Cluster for extensive psychological and/or psychiatric treatment).

#### Facilitators and treatment fidelity

METRA was delivered in Arabic by a social worker based at a local NGO specialising in the delivery of mental health programs. METRA and TAU were delivered by different NGOs and social workers. The social worker delivering METRA received four hours of METRA training in Arabic provided by two clinical psychologists. Over the course of METRA, the facilitator was able to access psychologists daily if needed regarding the delivery of METRA, managing participant distress, or regarding self-care. Participants were requested to not discuss the treatment with others. The clinical psychologists routinely monitored sessions and delivery by observing sessions and discussing content and delivery with the facilitator.

#### Feasibility and acceptability

The feasibility of recruitment was assessed by determining the number of adolescents who were approached and agreed to participate in METRA. We assessed the acceptability of intervention by measuring loss to follow-up. We determined the acceptability of treatment based on the number of METRA sessions attended. Following METRA and at follow-up, we conducted 15 interviews with adolescents and the facilitator to gain feedback on METRA.

#### Data analysis plan

We analysed data using Stata, version 17 (StataCorp LLC, College Station, Texas, USA). Analyses were on intent-to-treat principle, with all randomised participants analysed in their allocation condition. We examined the primary objective, and secondary objectives two and three using linear mixed effects models with intervention type, time, and intervention by time interaction as fixed factors. We modelled repeated assessments of individuals as random intercepts. Of primary interest in these analyses was the intervention by time interaction, which compared the levels of change over time in outcomes of the METRA and TAU groups. Linear mixed effects modelling analyses are robust to missing data; cases with missing data at later time points were retained in the analyses. We conducted analyses using all data points for the participants who were randomised (*i.e.* intent to treat). To investigate the mechanisms mediating any treatment effects (secondary objective three), we conducted structural equation modelling [52].

#### Cost, cost-effectiveness, and affordability

We costed both the delivery of METRA and TAU from a provider perspective, including only costs that would be incurred in a non-research setting, where we assumed METRA would be delivered by an NGO with existing facilities in the settlement or host community but supervised by international mental health experts. For METRA, we used audited project expenditure reports to identify relevant startup and implementation fiscal costs (training, staff time, materials), then costed them using an ingredients approach. For TAU, we assumed no startup costs since this treatment was a continuation of ongoing service provision and estimated only implementation costs (staff time) based on data collected for a sample of TAU participants (n = 35) on the number of sessions attended and time per session. We summed all relevant costs (Appendix S1 in the **Online Supplementary Document**) and divided by the number of participants in each group, using an intention-to-treat protocol. We reported total costs (separately for startup and implementation), cost per patient and cost per point decrease in PTSD and depression symptoms. We did not discount costs or effects because none occur in the future, given the less than one-year period of the intervention and assessment [53]. All costs are reported in 2023 USD. We assessed the potential affordability of using METRA in humanitarian settings using data from 19 LMICs reporting the financial resources required and those available to support people targeted for health sector assistance in various humanitarian settings [54]. We compared these estimates against the total costs of METRA, assuming 20% of adolescents in the population targeted for assistance would need mental health interventions [55].

### RESULTS

#### Group characteristics

The overall sample size for primary analyses included 67 adolescents in the METRA group and 65 adolescents in the TAU group. When we also conducted the below analyses, including age, gender and location of birth as covariates, the effect sizes remained largely consistent with the unadjusted results, showing minimal confounding influence (**Table 1**).

**Table 1:** Demographic variables*

Variables	METRA	TAU	Statistics	*P*-value
Age in years, x̄ (SD)	12.93 (1.93)	13.43 (1.96)	1.49†	0.07
Gender			0.75‡	0.39
*Male*	37	31		
*Female*	30	34		
Location of birth			28.75§	<0.001
*Kirkuk*	50	51		
*Salahuddine*	5	0		
*Anbar*	0	10		
*Tikrit*	9	1		
*Baghdad*	1	3		
*Arbil*	2	0		
Required to leave place of birth			0.75‡	0.39
*No*	48	42		
*Yes*	19	23		
If required to leave place of birth, moved to¶			<0.01‖	0.96
*Kirkuk*	15	18		
Other**	4	5		
Adults in family, x̄ (SD)	2.75 (1.04)	2.29 (0.74)	2.89†	<0.01
Children in family, x̄ (SD)	2.87 (1.92)	2.75 (1.60)	0.36†	0.36

METRA – MEmory Training for Recovery-Adolescent, SD – standard deviation, TAU – treatment as usual, x̄ – mean

*Presented as n unless specified otherwise.

†T-statistic (n = 130).

‡χ^2^ test (n = 132).

§Fisher exact test.

¶Not all participants reported where they had moved to.

‖χ^2^ test (n = 42).

**Other includes Anbar, Erbil, Mosul and Turkey.

#### Primary objective: PTSD and depression symptomatology post-intervention

**Figure 2:**
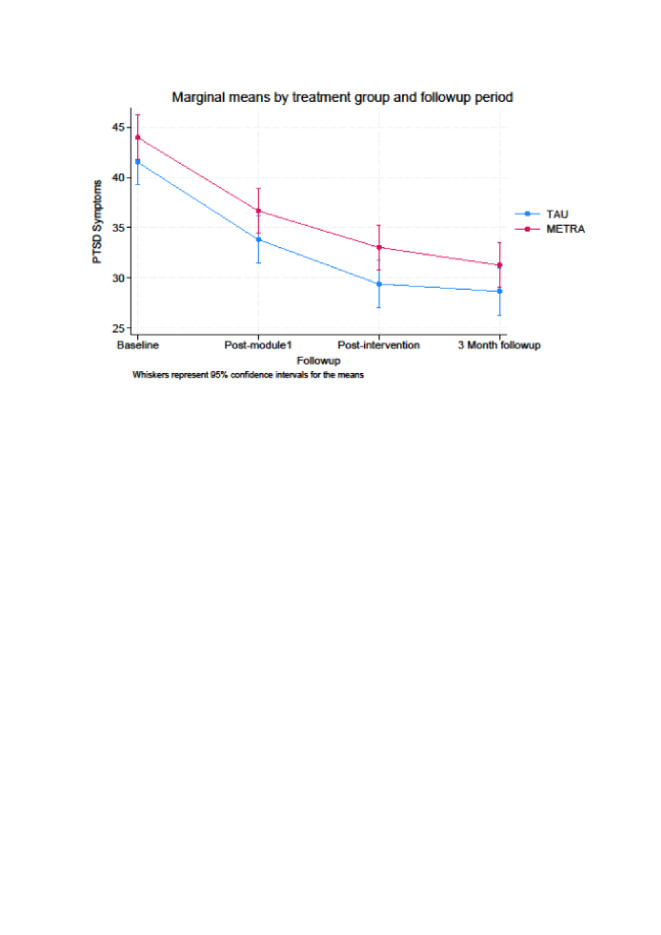
Marginal means by treatment group and follow-up period for posttraumatic stress disorder (PTSD) symptoms.

**Figure 3:**
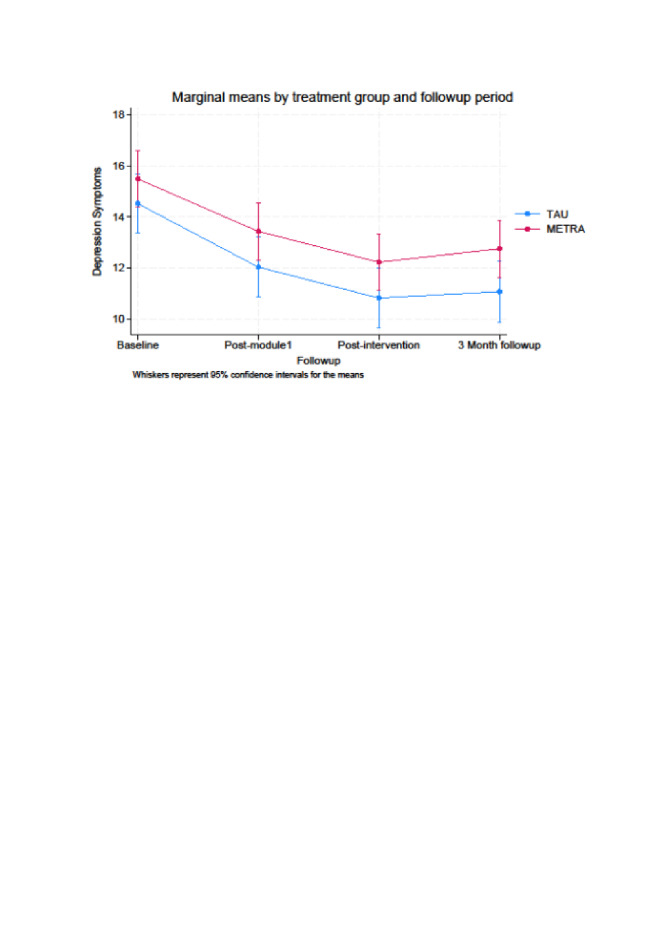
Marginal means by treatment group and follow-up period for depression symptoms.

When comparing post-intervention data to baseline data, we found for PTSD and depression symptoms significant time main effects (*P* < 0.001) (**Figure 2****,** **Figure 3****,** **Table 2**). At post-intervention, the METRA group had a 10.96-point (95% confidence interval (CI) = –13.82, –8.09) decrease in PTSD symptoms from baseline, while TAU had a 12.13-point (95% CI = –14.91, –9.34) decrease. The METRA group had a 3.27-point (95% CI = –4.67, –1.87) decrease in depression symptoms from baseline, while the TAU group had a 3.67-point (95% CI = –5.12, –2.22) decrease. Thus, in both groups, there were improvements in PTSD and depression scores. However, contrary to our hypothesis, there was no evidence to indicate the two groups differed significantly over time, as the group over time interactions were not significant for PTSD symptoms (*P* = 0.61) or depression symptoms (*P* = 0.71).

**Table 2:** Outcome measures by condition and time point*

Outcome measure	Baseline	Post-Module 1	Post-intervention	Follow-up
PTSD symptoms				
*METRA*	44.00 (41.76, 46.24)	36.69 (34.44, 38.93)	33.04 (30.80, 35.29)	31.27 (29.03, 33.51)
*TAU*	41.54 (39.26, 43.82)	33.84 (31.49, 36.19)	29.40 (27.03, 31.77)	28.65 (26.28, 31.02)
Depression symptoms				
*METRA*	15.49 (14.37, 16.61)	13.43 (12.31, 14.55)	12.22 (11.11, 13.34)	12.75 (11.63, 13.86)
*TAU*	14.52 (13.35, 15.70)	12.03 (10.86, 13.20)	10.82 (9.64, 12.00)	11.07 (9.89, 12.25)
Anxiety symptoms				
*METRA*	12.13 (10.97, 13.30)	11.99 (10.82, 13.15)	11.94 (10.77, 13.11)	10.73 (9.57, 11.89)
*TAU*	12.90 (11.68, 14.12)	12.52 (11.31, 13.74)	10.97 (9.74, 12.20)	11.12 (9.89, 12.35)
Psychiatric difficulties				
*METRA*	19.67 (18.65, 20.69)	20.84 (19.82, 21.86)	19.88 (18.86, 20.90)	19.75 (18.73, 20.77)
*TAU*	18.62 (17.55, 19.69)	19.10 (18.03, 20.17)	17.72 (16.64, 18.79)	17.68 (16.61, 18.76)

METRA – MEmory Training for Recovery-Adolescent, PTSD – posttraumatic stress disorder, TAU – treatment as usual

*Presented as marginal means and 95% CIs. Data presented is from the marginal means for the full model with interaction.

To assess potential subgroup effects based on gender and age, we conducted interaction tests between these variables and the main exposure variables (*i.e.* PTSD and depression symptoms). We found the results to be non-significant for PTSD (*P* = 0.41 for gender and *P* = 0.55 for age) and depression (*P* = 0.68 for gender and *P* = 0.43 for age).

#### Secondary objective: anxiety and psychiatric symptoms post-intervention

At post-intervention, compared to baseline, the main time effect was significant for anxiety symptoms (*P* < 0.03) (**Table 2**; Figure S1 in the **Online Supplementary Document**). The METRA group had a 0.17-point (95% CI = –1.72, 1.37) decrease in anxiety symptoms from baseline, while the TAU group had a 1.95-point (95% CI = –3.44, –0.46) decrease. Therefore, METRA had little impact on anxiety symptoms, and TAU showed a more substantial decrease in anxiety symptoms. It is worth noting that the confidence interval for the METRA group included zero, while the confidence interval for the TAU group did not include zero. This indicates that the TAU group experienced a statistically significant reduction in anxiety, but the METRA group did not. However, our results do not allow us to conclude that this between-group difference was significant as the group over time interaction was not significant (*P* = 0.15). There was no significant time main effect (*P* = 0.24) or group over time interaction (*P* = 0.30) for psychiatric difficulties (**Table 2**; Figure S2 in the **Online Supplementary Document**). Post-hoc power calculations revealed that this study was inadequately powered to detect the minimum change scores over time for both secondary outcomes (anxiety power = 48.9%, psychiatric difficulties power = 45.0%) with the current effect size observed.

#### Secondary objective: three-month follow-up analyses

When comparing the three-month follow-up data to the baseline data, all time main effects were significant for PTSD (*P* < 0.001), depression (*P* < 0.001), and anxiety (*P* = 0.04). However, the group over time interactions were not significant for PTSD (*P* = 0.95), depression (*P* = 0.54), or anxiety (*P* = 0.75). The METRA group had an 12.73-point (95% CI = –15.60, –9.87) decrease in PTSD symptoms (TAU = 12.88; 95% CI = –15.66,–10.09), 2.75-point (95% CI = –4.15,–1.35) decrease in depression (TAU = 3.42; 95% CI = –4.87,–1.97), and 1.40-point (95% CI = –2.94, –0.14) decrease in anxiety (TAU = 1.80; 95% CI = –3.30, –0.31). There was no significant time main effect (*P* = 0.23) or group over time interaction (*P* = 0.34) for psychiatric difficulties.

#### Secondary objective: mediation analyses

There was a moderate mediation effect of rumination for PTSD and depression symptoms and a low mediation effect of avoidance for PTSD and depression symptoms (**Table 3**). Most of the effect of METRA on reductions in symptoms of PTSD and depression were attributed through direct effect, with the ratio of indirect effects for rumination (ratio = 0.58, 0.71) and avoidance (ratio = 0.09, 0.10). The direction of effect through mediation variables was also opposite as compared to direct effects. Thus, the effect of METRA on the outcomes due to the indirect effects was smaller as compared to the direct effect.

**Table 3:** Mediating effects of rumination and cognitive avoidance on PTSD and depression symptomatology from baseline to follow-up

Effects	Coefficient	SE	Z-score	*P*-value	Standardised coefficient	Ratio of indirect *vs.* direct effect
PTSD baseline						
*Direct*	2.86	1.88	1.53	0.127	0.12	
*Indirect through rumination*	–1.65	0.95	–1.73	0.083	–0.07	–0.58
*Total*	1.21	2.06	0.59	0.557	0.05	
PTSD follow up						
*Direct*	1.35	2.05	0.66	0.511	0.06	
*Indirect through cognitive avoidance*	–0.13	0.28	–0.48	0.629	–0.01	–0.10
*Total*	1.21	2.06	0.59	0.557	0.05	
Depression baseline						
*Direct*	1.24	1.09	1.14	0.253	0.09	
*Indirect through rumination*	–0.88	0.51	–1.72	0.086	–0.07	–0.71
*Total*	0.36	1.17	0.31	0.756	0.03	
Depression follow-up						
*Direct*	0.34	1.17	0.29	0.775	0.03	
*Indirect through cognitive avoidance*	0.03	0.08	0.37	0.709	0.00	0.09
*Total*	0.36	1.17	0.31	0.756	0.03	

PTSD – posttraumatic stress disorder, SE – standard error

#### Secondary objective: cost, cost-effectiveness, and affordability analysis

Startup costs for METRA totalled USD 942. The total implementation cost of METRA was estimated to be USD 3340, or USD 33 per group session. Given 67 participants, the cost per participant was USD 50, or USD 5 per participant per session (Table S2 in the **Online Supplementary Document**). Implementation costs of TAU were estimated at USD 6974, based on an average of 6.32 sessions per participant and a session length of 50 minutes (Table S3 in the **Online Supplementary Document**). Thus, for the participants in the trial, METRA total implementation costs were less than those of TAU. The cost per point decrease in PTSD symptoms was USD 305 for METRA and USD 575 for TAU, and USD 1022 and USD 1900 per point decrease in depression symptoms for METRA and TAU, respectively (Figure S3 in the **Online Supplementary Document**).

We estimate rolling out METRA to 20% of adolescents targeted for health interventions (*i.e.* those estimated to require mental health services) in 19 humanitarian settings would represent 5% of current financial needs for the entire health sector and 39% of available funding for health, likely making it unaffordable at scale given the current funding landscape (Table S4 in the **Online Supplementary Document**).

#### Secondary objective: feasibility and acceptability

All participants allocated to the METRA group commenced METRA. No participants dropped out of METRA. For those in the METRA group, there were no important harms or unintended effects reported. Qualitative feedback was positive, noting participants enjoyed METRA and benefited from it. Several adolescents wanted their stories shared, and the community requested another course of METRA as there are so few mental health programs available. Points for improvement included the time specified for the initial METRA sessions not appearing long enough and the writing of memories being difficult for some youth with literacy concerns and for some younger adolescents.

## DISCUSSION

This study investigated the efficacy of METRA in addressing psychiatric concerns among adolescents in Iraq. Those who participated in METRA had significant reductions in PTSD and depression symptoms at post-intervention, and PTSD, depression and anxiety symptoms at three-month follow-up. However, there was no evidence these improvements were superior to the symptom improvements gained by the TAU group. Thus, we cannot conclude in this study that METRA was superior to TAU. Additionally, there was a moderate mediation effect of rumination for PTSD and depression, suggesting that changes in rumination may play a role in symptom improvement. This aligns with research indicating that reduced memory specificity and the remembering of trauma is associated with rumination, a factor that maintains PTSD and depression [22].

In the previous Afghanistan studies, METRA was found to improve symptoms of PTSD and depression, and these improvements did exceed those observed in the control groups [26,27]. In the current study, while we also observed METRA improved PTSD and depression symptoms, symptom improvements did not exceed that observed in the TAU group; we found no evidence to suggest METRA was superior to TAU. The difference between the Afghanistan studies and the current study may reflect the choice of control conditions. In the Afghanistan studies, the conditions were less ‘active’ (puberty health group [26]; study skill group [27]) and not trauma-focused. In this study, TAU was trauma-focused, aligning with current recommended evidence-based interventions for adolescents in LMICs [8], and delivered by skilled social workers and psychologists. Thus, TAU may already be an effective treatment, and both interventions may, therefore, lead to improvements in symptomatology. Modifications may be needed to METRA to further improve its benefits, including the potential need for additional sessions, greater spacing between sessions, longer periods of trauma writing in Module 2, or METRA benefiting older youth; participants in the Afghanistan studies were older (x̄ = 15.96 years (SD = 1.97) [27]; x̄ = 16.69 years (SD = 1.24) [28]) than in this study (x̄ = 13.17 years; SD = 1.95) and feedback from facilitators noted younger participants found METRA more challenging.

Potential mechanisms of action for METRA are improvements in rumination and memory specificity, and exposure work leading to reductions in subjective distress and integration of the trauma memory [22,25,50,51]. Given TAU was also trauma-focused, TAU may also target these mechanisms, which may contribute to the group x time interactions being non-significant. Further research is needed to explore the mechanisms of change of METRA. It is also important to note that while METRA and TAU were effective in reducing PTSD and depression in this context, the findings may not be generalised to other humanitarian contexts. While the challenges (*e.g.* long-term conflict, socio-political instability) facing Kirkuk are shared by many humanitarian settings and thus the findings have potential relevance to similar settings, particularly in the Middle East, interventions effective in one humanitarian context may not be adequate for delivery in other contexts and may need to be tailored for different sociocultural settings [8].

METRA was initially designed to be implemented over a ten-week period – as this aligns with previous MEST [19,20] and written exposure protocols [51]. However, as in the Afghanistan studies, practitioners and community in Kirkuk decided it was more appropriate for logistical and security reasons to deliver METRA over a fortnight, which may have influenced findings. However, it does highlight that in humanitarian contexts, it may be important for interventions to be delivered intensively over shorter timeframes. Emerging research is indicting that shorter intensive delivery of exposure treatments are non-inferior to standard (10-week) approaches [56].

Although METRA participants attended more sessions (10 *vs.* 6.32 on average for TAU) of a longer duration (60 *vs.* 50 minutes), METRA sessions were less expensive to implement on a per participant basis given the group setting, even when also considering the initial training and materials translation. Given this study’s findings on effectiveness, countries may be hesitant to replace TAU with METRA, though METRA still offers distinct benefits over TAU, notably that it was able to be readily administered in groups by health professionals with minimal additional training and supervision and at a lower cost.

However, when compared against existing mental health spending in humanitarian settings, our cost estimates suggest that METRA is unlikely to be affordable if rolled out at scale to the 20% of adolescents estimated to need mental health services in these settings. This may speak more to the affordability of mental health interventions and/or humanitarian funding availability in general than to METRA, as similar interventions targeting PTSD and depression symptoms amongst humanitarian populations found a cost per participant of USD 26–175, making them equally unlikely to be affordable at scale [32,33,35,37]. Although not affordable at scale, METRA is likely a more affordable alternative to the current mental health interventions offered by NGOs and governments in humanitarian settings. Future studies could consider modifications to METRA that further reduce costs and improve scalability, such as delivery to larger groups (*e.g.* through schools), examining the necessity of both modules and number of sessions for symptom improvement, and considering the potential of online delivery – there is growing evidence-base for digital interventions in LMICs [7].

We chose not to use traditional economic evaluation methods which consider cost per disability-adjusted life-years averted and wider societal benefits beyond the primary and secondary outcomes captured in our study, such as gains in productivity and employment and potential reduction in the cost of social support services [30]. These methods may not be appropriate in humanitarian settings, produce results that are difficult for donors, NGO implementers and government counterparts to interpret and give no indication about whether the intervention is financially feasible in the setting [57,58]. We believe considering affordability for service providers in simple terms as we have done, on a per participant basis and against available funding, is the most useful approach and can help support advocacy efforts aimed at garnering more funding for these interventions.

In terms of feasibility and acceptability, all participants allocated to METRA commenced the intervention, and no participants dropped out, which is important given that up to 50% of patients drop out of PTSD interventions [59]. Qualitative feedback from youth who completed METRA was positive, noting satisfaction and benefit from METRA. In further considering the implementation of METRA it is worth considering extra time for the initial sessions and METRA may be more suitable for older adolescents (≥12 years). It is also worth noting the ethical considerations of conducting this study. Several families requested that their children who were just under 10 or the participant’s siblings participate in METRA. Due to limited services in Kirkuk, we offered METRA to these youth by adding additional groups at the end that were not included in the study. Despite initial concerns about Module 2’s trauma focus, both families and the NGO reported that distress was well-managed within METRA and reduced throughout the module. To ensure METRA's sustainability, all materials have been made freely available in Arabic and Kurdish. During the study, a brief period of conflict in Kirkuk [60] required us to pause the study for a few days for safety reasons. Lastly, youth requested that the findings be shared through a graphic novel and animation, which the team is currently developing to ensure community input into the research outputs.

### Limitations

This study has several limitations. First, our results did not allow us to conclude that this difference between the two groups over time was statistically significant, as the group x time interactions were not significant. Our post-hoc power calculations showed that this study was inadequately powered to detect the minimum change scores over time for both secondary outcomes (anxiety, psychiatric difficulties) and the observed differences were small and may not be clinically significant. Therefore, a larger sample size is needed to explore this further in future studies. Second, the lack of long-term follow-up precludes us from knowing whether METRA treatment gains were maintained beyond three months post-treatment. Future studies should include longer (*e.g.* six, 12, 24-month) follow-up periods. This important as adolescents in humanitarian settings often continue to face human right violations and periods of conflict, which can impact mental health. Hence, it is important to examine whether the skills learnt in METRA can mitigate any of these mental health challenges. Third, we did not include measures assessing memory specificity (*e.g.* Autobiographical Memory Test) [22] or subjective units of distress during the trauma writing [51,61], which could provide further details regarding mechanisms of change. Investigating these mechanisms is important as it may shed light on the durability of METRA’s benefits. Fourth, due to the nature of the intervention, we could not blind participants and facilitators, which may have biased the trial and findings by influencing facilitators' delivery of METRA, participants’ behaviour in the trial, and responses on outcome measures. Fifth, the SDQ only had acceptable internal consistency. Thus, the findings relating to the SDQ should be interpreted with caution. Sixth, as the study was conducted in Kirkuk, caution needs to be heeded if considering METRA for other parts of Iraq or other humanitarian regions. Seventh, the spacing of METRA in this trial differed to the spacing of sessions delivered in the Afghanistan trials, which may have influenced findings. Eighth, future studies would benefit in recording each of the METRA intervention sessions to ensure treatment fidelity. Ninth, the cost-analysis excluded METRA development costs, since it was a pre-existing intervention adapted to our setting through the translation of materials. Others wishing to utilize METRA in their own settings will likely incur similar translation costs. Likewise, we have excluded any startup costs related to TAU since mental health services were already available. Finally, future studies could examine the utility of Module 1 in benefiting treatment effects observed in Module 2, and whether there is a need to deliver both modules.

## CONCLUSIONS

We found adolescents in Iraq receiving METRA had significant improvements in PTSD and depression. However, these improvements did not exceed those observed in TAU group. Our findings suggest that METRA may be a promising intervention for adolescents in humanitarian contexts and highlight the need for more research in this area of global mental health. The study has implications for policy and practice. It underscores the need for global health to prioritise mental health interventions for adolescents in humanitarian contexts and calls for increased international funding for such interventions. Nearly 95% of community youth screened for this study met the criteria for PTSD and/or depression, highlighting the urgent need for mental health support in Kirkuk and the importance of Iraq prioritising adolescent mental health in policy and funding [12]. The study also shows the importance of LMICs allocating a significant proportion of the health budget to adolescent mental health and the findings indicated interventions can be effectively delivered by community health facilitators and NGOs [6]. Finally, the study emphasises the need for more low-cost, low-intensity interventions for complex disorders like PTSD that can be implemented in LMICs [7,9].

## Additional material


Online Supplementary Document

## References

[1] United Nations International Children’s Emergency Fund. Adolescents. 2024. Available: https://data.unicef.org/topic/adolescents/overview/. Accessed: 14 May 2024.

[2] Jones N, Pincock K, Abu Hamad B, editors. Adolescents in humanitarian crisis. Displacement, Gender and Social Inequalities. London, UK: Routledge; 2021.

[3] Ahmadi SJ Jobson L Earnest A McAvoy D Musavi Z Samim N Prevalence of Poor Mental Health Among Adolescents in Kabul, Afghanistan, as of November 2021. JAMA Netw Open. 2022;5:e2218981. 10.1001/jamanetworkopen.2022.18981 35737391 PMC9226996

[4] Devonald M Vintges J Jones N Supporting adolescent mental health in humanitarian settings: To what extent do interventions consider climate change and its intersectional impacts? Intervention (Amstelveen). 2022;20:81–97. 10.4103/intv.intv_31_21

[5] Singh NS DeJong J Popple K Undie CC El Masri R Bakesiima R Adolescent wellbeing in humanitarian and fragile settings: moving beyond rhetoric. BMJ. 2023;380:e068280. 10.1136/bmj-2021-068280 36940938 PMC10019456

[6] Ndetei DM Mutiso V Osborn T Moving away from the scarcity fallacy: three strategies to reduce the mental health treatment gap in LMICs. World Psychiatry. 2023;22:163–4. 10.1002/wps.21054 36640407 PMC9840495

[7] Wani C McCann L Lennon M Radu C Digital mental health interventions for adolescents in low- and middle-income countries: scoping review. J Med Internet Res. 2024;26:e51376. 10.2196/51376 39471371 PMC11558223

[8] Ribeiro WS Grande AJ Hoffmann MS Ziebold C McDaid D Fry A A systematic review of evidence-based interventions for child and adolescent mental health problems in low- and middle-income countries. Compr Psychiatry. 2023;121:152358. 10.1016/j.comppsych.2022.152358 36508775

[9] Alzaghoul AF McKinlay AR Archer M Post-traumatic stress disorder interventions for children and adolescents affected by war in low- and middle-income countries in the Middle East: Systematic review. BJPsych Open. 2022;8:e153. 10.1192/bjo.2022.552 35938530 PMC9380009

[10] Kumar M Bhat A Unützer J Saxena S Editorial: strengthening child and adolescent mental health (CAMH) services and systems in lower-and-middle-income countries (LMICs). Front Psychiatry. 2021;12:645073. 10.3389/fpsyt.2021.645073 33633617 PMC7901940

[11] United Nations High Commissioner for Refugees. Iraq situation: 2024 situation overview. 2024. Available: https://reporting.unhcr.org/operational/situations/iraq-situation#:~:text=The%20needs%20remain%20high%20in,or%20to%20effective%20local%20integration. Accessed: 6 May 2024.

[12] Saied AA Ahmed SK Talib H Abdulqadir SO Omar RM Mental healthcare in Iraq - Time to be a priority. Asian J Psychiatr. 2023;84:103539. 10.1016/j.ajp.2023.103539 36989733

[13] Saied AA Ahmed SK Metwally AA Aiash H Iraq’s mental health crisis: a way forward? Lancet. 2023;402:1235–6. 10.1016/S0140-6736(23)01283-7 37805209

[14] Al Juboori R Violence and Child Mental Health Outcomes in Iraq: Mapping Vulnerable Areas. Psychiatry Int. 2024;5:39–52. 10.3390/psychiatryint5010004

[15] Shareef S A study on the human rights situation in Kirkuk. The Age of Human Rights Journal. 2023;2023:e7374. 10.17561/tahrj.v20.7374

[16] AlObaidi A Iraq: children’s and adolescents’ mental health under conditions of continuous turmoil. Int Psychiatry. 2011;8:4–5. 10.1192/S1749367600006135 31508062 PMC6735008

[17] Juengsiragulwit D Opportunities and obstacles in child and adolescent mental health services in low- and middle-income countries: a review of the literature. WHO South-East Asia J Public Health. 2015;4:110–22. 10.4103/2224-3151.206680 28607309

[18] Ahmadi SJ Musavi Z Samim N Sadeqi M Jobson L Investigating the feasibility, acceptability and efficacy of using modified-written exposure therapy in the aftermath of a terrorist attack on symptoms of posttraumatic stress disorder among Afghan adolescent girls. Front Psychiatry. 2022;13:826633. 10.3389/fpsyt.2022.826633 35463492 PMC9027104

[19] Neshat-Doost HT Dalgleish T Yule W Kalantari M Ahmadi SJ Dyregov A Enhancing autobiographical memory specificity through cognitive training: an intervention for depression translated from basic science. Clin Psychol Sci. 2013;1:84–92. 10.1177/2167702612454613

[20] Raes F Williams JM Hermans D Reducing cognitive vulnerability to depression: a preliminary investigation of MEmory Specificity Training (MEST) in inpatients with depressive symptomatology. J Behav Ther Exp Psychiatry. 2009;40:24–38. 10.1016/j.jbtep.2008.03.001 18407245

[21] Hitchcock C Nixon RD Weber N A review of overgeneral memory in child psychopathology. Br J Clin Psychol. 2014;53:170–93. 10.1111/bjc.12034 24921070

[22] Williams JM Barnhofer T Crane C Herman D Raes F Watkins E Autobiographical memory specificity and emotional disorder. Psychol Bull. 2007;133:122–48. 10.1037/0033-2909.133.1.122 17201573 PMC2834574

[23] Erten MN Brown AD Memory Specificity Training for Depression and Posttraumatic Stress Disorder: A Promising Therapeutic Intervention. Front Psychol. 2018;9:419. 10.3389/fpsyg.2018.00419 29666598 PMC5892288

[24] Ahmadi SJ Kajbaf MB Neshat Doost HT Dalgleish T Jobson L Mosavi Z The efficacy of memory specificity training in improving symptoms of post-traumatic stress disorder in bereaved Afghan adolescents. Intervention (Amstelveen). 2018;16:243–8. 10.4103/INTV.INTV_37_18

[25] Brewin CR The nature and significance of memory disturbance in posttraumatic stress disorder. Annu Rev Clin Psychol. 2011;7:203–27. 10.1146/annurev-clinpsy-032210-104544 21219190

[26] Ahmadi SJ Jobson L Musavi Z Rezwani SR Amini FA Earnest A Effect of the Memory Training for Recovery-Adolescent Intervention vs Treatment as Usual on Psychiatric Symptoms Among Adolescent Girls in Afghanistan: A Randomized Clinical Trial. JAMA Netw Open. 2023;6:e236086. 10.1001/jamanetworkopen.2023.6086 36995710 PMC10064255

[27] Ahmadi SJ Musavi Z Ahmadi S Masha S Muradi N Samim NU Examining MEmory Training for Recovery-Adolescent among Afghan adolescent boys: a pilot randomised controlled trial. Eur J Psychotraumatol. 2023;14:2251780. 10.1080/20008066.2023.2251780 37672117 PMC10484046

[28] Blanchet K, Roberts B. An evidence review of research on health interventions in humanitarian crises. Cardiff, UK: ELHRA: Enhancing learning and research for humanitarian purposes; 2015. Available: https://www.elrha.org/docs/document/evidence-review-22.10.15.pdf?file_url=document/j6fq10ehkd7mjfqonq84pkfo7o/-usgwp5dvbydv3flbn7izvh137e/original?content-type=application%2fpdf&name=evidence-review-22.10.15.pdf. Accessed: 22 May 2024.

[29] Miles T, Powell T, Lough BJ. Mental Health Research in Humanitarian and Development Settings. 2023. Available: https://www.researchgate.net/publication/377689815_Mental_Health_Research_in_Humanitarian_and_Development_Settings. Accessed: 10 April 2025.

[30] Tol WA Le PD Harrison SL Galappatti A Annan J Baingana FK Mental health and psychosocial support in humanitarian settings: research priorities for 2021-30. Lancet Glob Health. 2023;11:e969–75. 10.1016/S2214-109X(23)00128-6 37116530 PMC10188364

[31] Rathod S Pinninti N Irfan M Gorczynski P Rathod P Gega L Mental Health Service Provision in Low- and Middle-Income Countries. Health Serv Insights. 2017;10:1178632917694350. 10.1177/1178632917694350 28469456 PMC5398308

[32] Hamdani SU Huma ZE Rahman A Wang D Chen T van Ommeren M Cost-effectiveness of WHO Problem Management Plus for adults with mood and anxiety disorders in a post-conflict area of Pakistan: randomised controlled trial. Br J Psychiatry. 2020;217:623–9. 10.1192/bjp.2020.138 32720628

[33] Abi Hana R Abi Ramia J Burchert S Carswell K Cuijpers P Heim E Cost-Effectiveness of Digital Mental Health Versus Usual Care During Humanitarian Crises in Lebanon: Pragmatic Randomized Trial. JMIR Ment Health. 2024;11:e55544. 10.2196/55544 38810255 PMC11170045

[34] Park AL Waldmann T Kösters M Tedeschi F Nosè M Ostuzzi G Cost-effectiveness of the Self-Help Plus Intervention for Adult Syrian Refugees Hosted in Turkey. JAMA Netw Open. 2022;5:e2211489. 10.1001/jamanetworkopen.2022.11489 35536574 PMC9092202

[35] McBain RK Salhi C Hann K Salomon JA Kim JJ Betancourt TS Costs and cost-effectiveness of a mental health intervention for war-affected young persons: decision analysis based on a randomized controlled trial. Health Policy Plan. 2016;31:415–24. 10.1093/heapol/czv078 26345320 PMC5007601

[36] McDaid D, Park A. Economic modelling of scaling up implementation. London, UK: London School of Economics, Project STRENGHTS; 2022. Available: https://strengths-project.eu/wp-content/uploads/2023/03/STRENGTHS-D7.3-Economic-modelling-of-scaling-up-implementation.pdf. Accessed: 10 April 2025.

[37] Zeng W Samaha H Yao M Ahuka-Mundeke S Wilkinson T Jombart T The cost of public health interventions to respond to the 10th Ebola outbreak in the Democratic Republic of the Congo. BMJ Glob Health. 2023;8:e012660. 10.1136/bmjgh-2023-012660 37848269 PMC10583089

[38] Perrin S Meiser-Stedman R Smith P The children’s revised impact of event scale (CRIES): validity as a screening instrument for PTSD. Behav Cogn Psychother. 2005;33:487–98. 10.1017/S1352465805002419

[39] Angold A Costello EJ Messer SC Pickles A Development of a short questionnaire for use in epidemiological studies of depression in children and adolescents. Int J Methods Psychiatr Res. 1995;5:237–49.

[40] Veronese G Pepe A Measuring Traumatic Reactions in Palestinian Children: A Psychometric Assessment of the Children Revised Impact of Event Scale-Arabic Version (CRIES-13A). Child Psychiatry Hum Dev. 2022;53:16–26. 10.1007/s10578-020-01113-2 33385256

[41] Al-Musawy J Husian M Hussein H Nashtar S Kadhum A Kadhum H Posttraumatic stress reactions among school children in Iraq. Azerbaijan Medical Journal. 2022;62:3421–32.

[42] Tavitian L Atwi M Bawab S Hariz N Zeinoun P Khani M The Arabic Mood and Feelings Questionnaire: psychometrics and validity in a clinical sample. Child Psychiatry Hum Dev. 2014;45:361–8. 10.1007/s10578-013-0406-6 24081605

[43] Reynolds CR Richmond BO What I think and feel: a revised measure of children’s manifest anxiety. J Abnorm Child Psychol. 1978;6:271–80. 10.1007/BF00919131 670592

[44] Freh FM PTSD, depression, and anxiety among young people in Iraq one decade after the American invasion. Traumatology (Tallahass Fla). 2016;22:56–62. 10.1037/trm0000062

[45] Goodman R Meltzer H Bailey V The Strengths and Difficulties Questionnaire: a pilot study on the validity of the self-report version. Eur Child Adolesc Psychiatry. 1998;7:125–30. 10.1007/s007870050057 9826298

[46] Alborz A, Al-Hashemy J, Al-Obaidi K, Brooker E, Miles S, Penn H, et al. A study of mainstream education opportunities for disabled children and youth and early childhood development in Iraq. A report to UNICEF. Manchester, UK: University of Manchester; 2011. Available: https://pure.manchester.ac.uk/ws/portalfiles/portal/36763409/FULL_TEXT.PDF. Accessed: 10 April 2025.

[47] Roelofs J Rood L Meesters C te Dorsthorst V Bögels S Alloy LB The influence of rumination and distraction on depressed and anxious mood: a prospective examination of the response styles theory in children and adolescents. Eur Child Adolesc Psychiatry. 2009;18:635–42. 10.1007/s00787-009-0026-7 19415414 PMC2832856

[48] Sexton KA Dugas MJ The Cognitive Avoidance Questionnaire: validation of the English translation. J Anxiety Disord. 2008;22:355–70. 10.1016/j.janxdis.2007.04.005 17544253

[49] Kalantari M Yule W Dyregrov A Neshatdoost H Ahmadi SJ Efficacy of writing for recovery on traumatic grief symptoms of Afghani refugee bereaved adolescents: a randomized control trial. Omega (Westport). 2012;65:139–50. 10.2190/OM.65.2.d 22953510

[50] Sloan DM Marx BP Lee DJ Resick PA A Brief Exposure-Based Treatment vs Cognitive Processing Therapy for Posttraumatic Stress Disorder: A Randomized Noninferiority Clinical Trial. JAMA Psychiatry. 2018;75:233–9. 10.1001/jamapsychiatry.2017.4249 29344631 PMC5843538

[51] Sloan DM, Marx BP. Written Exposure Therapy for PTSD: A Brief Treatment Approach for Mental Health Professionals. Washington, D.C., USA: American Psychological Association; 2019.

[52] Kraemer HC Wilson GT Fairburn CG Agras WS Mediators and moderators of treatment effects in randomized clinical trials. Arch Gen Psychiatry. 2002;59:877–83. 10.1001/archpsyc.59.10.877 12365874

[53] Drummond MF, Sculpher MJ, Torrance GW, O’Brien BJ, Stoddart GL. Methods for the economic evaluation of health care programme. 3rd ed. Oxford, UK: Oxford University Press; 2005.

[54] Humanitarian Action. Inter-agency plans 2024. 2024. Available: https://humanitarianaction.info/. Accessed: 3 May 2024.

[55] World Health Organization. Providing mental health support in humanitarian emergencies: an opportunity to integrate care in a sustainable way. 2021. Available: https://www.who.int/news-room/feature-stories/detail/providing-mental-health-support-in-humanitarian-emergencies-an-opportunity-to-integrate-care-in-a-sustainable-way. Accessed: 15 April 2025.

[56] Dell L Sbisa AM Forbes A O’Donnell M Bryant R Hodson S Effect of massed v. standard prolonged exposure therapy on PTSD in military personnel and veterans: a non-inferiority randomised controlled trial. Psychol Med. 2023;53:4192–9. 10.1017/S0033291722000927 35440345 PMC10317798

[57] Woods B Revill P Sculpher M Claxton K Country-Level Cost-Effectiveness Thresholds: Initial Estimates and the Need for Further Research. Value Health. 2016;19:929–35. 10.1016/j.jval.2016.02.017 27987642 PMC5193154

[58] Puett C Assessing the cost-effectiveness of interventions within a humanitarian organisation. Disasters. 2019;43:575–90. 10.1111/disa.12344 31012136 PMC6850649

[59] Schottenbauer MA Glass CR Arnkoff DB Tendick V Gray SH Nonresponse and dropout rates in outcome studies on PTSD: review and methodological considerations. Psychiatry. 2008;71:134–68. 10.1521/psyc.2008.71.2.134 18573035

[60] Human Rights Watch. Iraq: Security forces open fire on Kirkuk protesters. 8 September 2023. Available: https://www.hrw.org/news/2023/09/08/iraq-security-forces-open-fire-kirkuk-protesters. Accessed: 15 April 2025.

[61] Li M Wang B Chen Q Gao D Zang Y Written exposure therapy and app-delivered mindfulness-based meditation for PTSD and subthreshold PTSD in China: Design of a randomized controlled trial. Contemp Clin Trials Commun. 2021;22:100729. 10.1016/j.conctc.2021.100729 34007950 PMC8111261

