# Supplementary material for: Randomised clinical trial investigating memory training for recovery-adolescents in addressing psychiatric concerns among adolescents in Iraq
Source: J Glob Health. 2025 May 5;15:04111. doi: 10.7189/jogh.15.04111 (PMC12209735; doi:10.7189/jogh.15.04111)
Supplement: Online Supplementary Document [file jogh-15-04111-s001.pdf]

Supplement to: Jobson L, Malallah H, Ahmadi SJ, McAvoy D, Earnest A, Vaughan K, Berzenji SL, Mohammad S, Berzengi A. Randomised clinical trial investigating memory training for recovery-adolescents in addressing psychiatric concerns among adolescents in Iraq. J Glob Health. 2025;15:04111.

CONSORT 2010 checklist of information to include when reporting a randomised trial*

Section/Topic	Item No	Checklist item	Reported on page No
**Title and abstract**			
	1a	Identification as a randomised trial in the title	1
	1b	Structured summary of trial design, methods, results, and conclusions (for specific guidance see CONSORT for abstracts)	2
**Introduction**			
Background and objectives	2a	Scientific background and explanation of rationale	3-7
	2b	Specific objectives or hypotheses	6-7
**Methods**			
Trial design	3a	Description of trial design (such as parallel, factorial) including allocation ratio	7
	3b	Important changes to methods after trial commencement (such as eligibility criteria), with reasons	N/A
Participants	4a	Eligibility criteria for participants	7
	4b	Settings and locations where the data were collected	7
Interventions	5	The interventions for each group with sufficient details to allow replication, including how and when they were actually administered	10-12
Outcomes	6a	Completely defined pre-specified primary and secondary outcome measures, including how and when they were assessed	8-10
	6b	Any changes to trial outcomes after the trial commenced, with reasons	N/A
Sample size	7a	How sample size was determined	7
	7b	When applicable, explanation of any interim analyses and stopping guidelines	N/A
Randomisation:			
Sequence generation	8a	Method used to generate the random allocation sequence	8
	8b	Type of randomisation; details of any restriction (such as blocking and block size)	8
Allocation concealment mechanism	9	Mechanism used to implement the random allocation sequence (such as sequentially numbered containers), describing any steps taken to conceal the sequence until interventions were assigned	8
Implementation	10	Who generated the random allocation sequence, who enrolled participants, and who assigned participants to interventions	8

Blinding	11a	If done, who was blinded after assignment to interventions (for example, participants, care providers, those assessing outcomes) and how	8
	11b	If relevant, description of the similarity of interventions	N/A
Statistical methods	12a	Statistical methods used to compare groups for primary and secondary outcomes	12-13
	12b	Methods for additional analyses, such as subgroup analyses and adjusted analyses	N/A
**Results**			
Participant flow (a diagram is strongly recommended)	13a	For each group, the numbers of participants who were randomly assigned, received intended treatment, and were analysed for the primary outcome	Figure 1
	13b	For each group, losses and exclusions after randomisation, together with reasons	Figure 1
Recruitment	14a	Dates defining the periods of recruitment and follow-up	8
	14b	Why the trial ended or was stopped	N/A
Baseline data	15	A table showing baseline demographic and clinical characteristics for each group	Table 1 and 2
Numbers analysed	16	For each group, number of participants (denominator) included in each analysis and whether the analysis was by original assigned groups	Figure 1
Outcomes and estimation	17a	For each primary and secondary outcome, results for each group, and the estimated effect size and its precision (such as 95% confidence interval)	14-16
	17b	For binary outcomes, presentation of both absolute and relative effect sizes is recommended	N/A
Ancillary analyses	18	Results of any other analyses performed, including subgroup analyses and adjusted analyses, distinguishing pre-specified from exploratory	N/A
Harms	19	All important harms or unintended effects in each group (for specific guidance see CONSORT for harms)	17
**Discussion**			
Limitations	20	Trial limitations, addressing sources of potential bias, imprecision, and, if relevant, multiplicity of analyses	21-22
Generalisability	21	Generalisability (external validity, applicability) of the trial findings	17-23
Interpretation	22	Interpretation consistent with results, balancing benefits and harms, and considering other relevant evidence	17-23
**Other information**			
Registration	23	Registration number and name of trial registry	7
Protocol	24	Where the full trial protocol can be accessed, if available	7
Funding	25	Sources of funding and other support (such as supply of drugs), role of funders	1

Citation: Schulz KF, Altman DG, Moher D, for the CONSORT Group. CONSORT 2010 Statement: updated guidelines for reporting parallel group randomised trials. BMC Medicine. 2010;8:18. © 2010 Schulz et al. This is an Open Access article distributed under the terms of the Creative Commons Attribution License (http://creativecommons.org/licenses/by/2.0), which permits unrestricted use, distribution, and reproduction in any medium, provided the original work is properly cited.

Supplementary Figure 1

Marginal Means by treatment group and follow-up period for anxiety symptoms

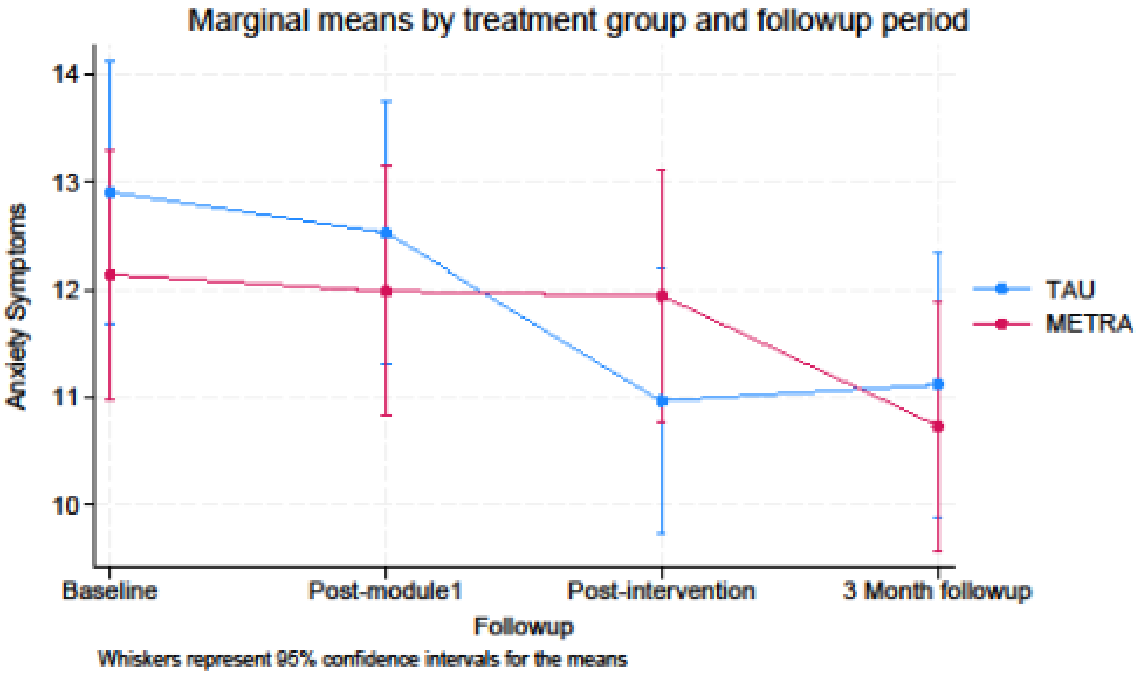

Supplementary Figure 2

Marginal Means by treatment group and follow-up period for psychiatric difficulties (SDQ)

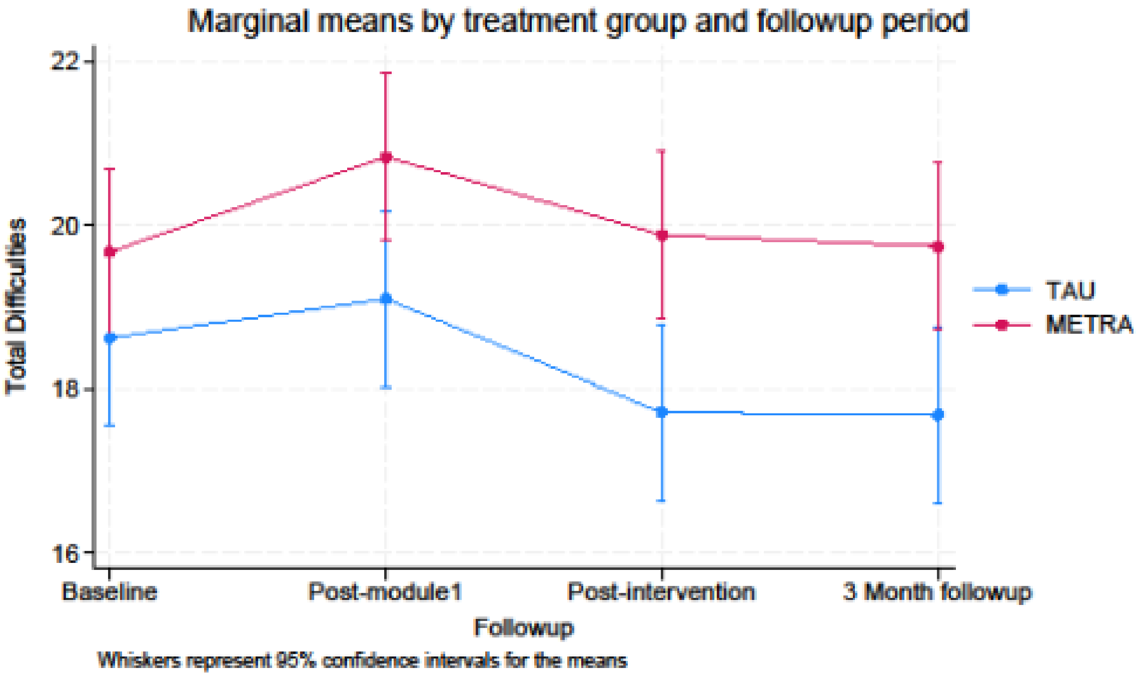

Excluded costs

Both TAU and METRA sessions were held at a local NGO's own premises, so we assumed no financial cost for the use of existing facilities. We also excluded indirect costs, such as administrative and accounting support staff, who support multiple projects. Finally, we excluded patient costs, given our focus on affordability of the interventions to implement in humanitarian settings.

Specific start-up costs for METRA

For startup costs, we included four hours of staff time for the initial training session (held over Zoom) and translation of existing METRA materials into Arabic. As implementation costs, we included staff time for preparation, delivery and clean-up of METRA sessions (1.5 hours/session for 10 groups of 10 sessions each), two hours of clinical supervision (2 supervisors and 1 social worker), and stationary for sessions estimated at $2/participant.

Specific implementation costs for TAU

For TAU, we estimated implementation costs, which were limited to staff time to deliver individual therapy sessions and refer necessary patients, we tracked the length and number of sessions for a sample of 35 patients, and calculated a weighted average per patient (6.32 sessions of 50.22 minutes each), which we applied to the remaining TAU patients (total 343.67 hours across 411 sessions). We assumed 1.9 referrals total based on one referral found in the sample, and 18.60 minutes total (10 minutes/referral). For supervision, we assumed the same total supervision time as METRA (total of 2 hours) for the social worker and her supervisor, a psychologist. Since we did not have access to the salary information for the social worker or her supervisor, we used locally validated estimates.

Supplementary Table 2

METRA startup and implementation costs (2023 US$)

		Description	Unit	No. units	Unit cost	Total cost
Startup costs						
	Zoom training: 2 clinical psychologist and social worker facilitator for 4 hours each	Clinical psychologist 1	Hours	4	43.47	173.87
		Clinical psychologist 2	Hours	4	36.78	147.12
		Social worker facilitator	Hours	4	20	80
	Translation of materials into Arabic		Each	1	541.50	541.50
	**Total startup costs**					**942.49**
Implementation costs						
	Session preparation, facilitation and clean-up: 1.5 hours/session x 10 sessions x 10 groups	Social worker facilitator	Hours	150	20	3,000
	Clinical supervision: 2 clinical psychologists and social worker facilitator for total of 2 hours each	Clinical psychologist 1	Hours	2	43.47	86.93
		Clinical psychologist 2	Hours	2	36.78	73.56
		Social worker facilitator	Hours	2	20	40
	Stationary: pens and paper for session activities	Materials per participant	Each	70	2	140
	**Total implementation costs**					**3,340.49**
	Implementation cost per participant (n=67)					49.86
	Implementation cost per group session (n=100)					33.40

Note: Clinical supervisors provided the four hour METRA training for the social workers. The social worker was able to access the clinical psychologists daily if needed regarding the delivery of METRA, managing participant distress, or regarding self-care.

Supplementary Table 3

TAU implementation costs (2023 US$)

		Description	Unit	No. units	Unit cost	Total cost
Implementation costs						
	Therapy sessions	Social worker	Hours	343.7	20	6,873.40
	Referrals	Social worker	Hours	0.3	20	6.20
	Clinical supervision: 1 psychologist and 1 social worker for 1 hour/week x 19 weeks	Psychologist	Hours	2	27	54
		Social worker	Hours	2	20	40
	**Total implementation costs**					**6,973.60**
	Implementation cost per participant (n=65)					107.29
	Implementation cost per individual session (n=411)					16.97

Supplementary Figure 3

Implementation costs for MEmory Training for Recovery – Adolescents (METRA) and Treatment-As-Usual (TAU)

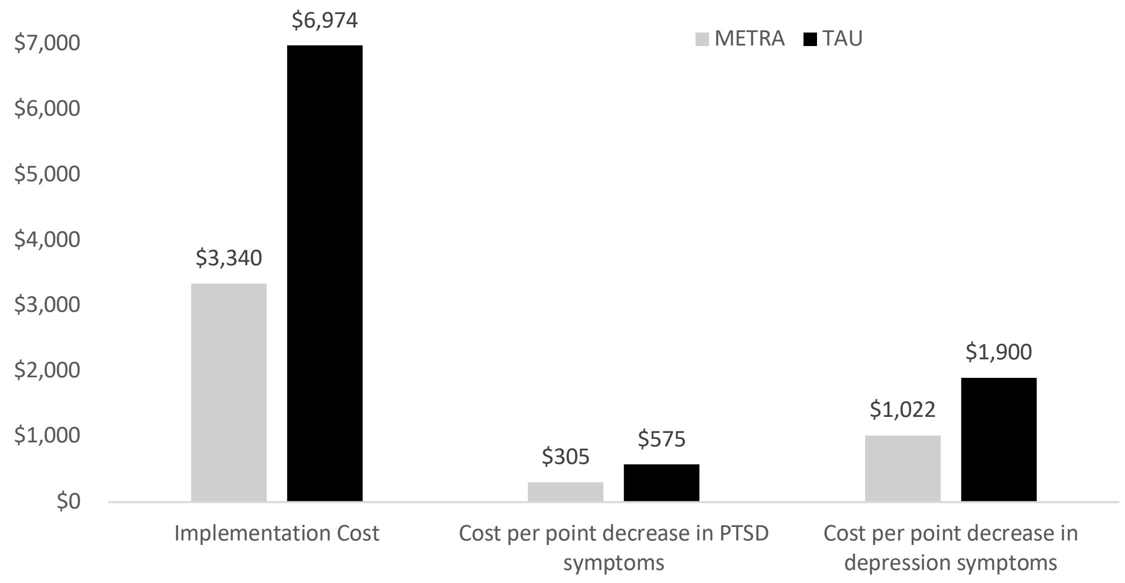

Supplementary Table 4

Humanitarian Response Plan (HRP) Country	People targeted (health services in general)	Est. number of persons in need of mental health services	Est. number of adolescents in need of METRA	Requirements for health (US$)	Funding coverage for entire HRP (incl. health)	Est. cost of METRA	METRA costs as % total health requirements
Afghanistan	12,800,000	2,560,000	409,600	367,000,000	15%	20,422,656	6%
Burkina Faso	1,600,000	320,000	51,200	55,700,000	8%	2,552,832	5%
Cameroon	1,100,000	220,000	35,200	30,500,000	12%	1,755,072	6%
Central African Republic	768,000	153,600	24,576	30,000,000	27%	1,225,359	4%
Chad	1,100,000	220,000	35,200	36,000,000	6%	1,755,072	5%
Colombia	784,000	156,800	25,088	48,600,000	13%	1,250,888	3%
Democratic Republic of the Congo	8,700,000	1,740,000	278,400	264,900,000	17%	13,881,024	5%
El Salvador	110,100	22,020	3,523	4,000,000	7%	175,667	4%
Ethiopia	6,700,000	1,340,000	214,400	187,300,000	9%	10,689,984	6%
Guatemala	304,000	60,800	9,728	5,800,000	10%	485,038	8%
Haiti	1,900,000	380,000	60,800	37,300,000	15%	3,031,488	8%
Honduras	339,000	67,800	10,848	1,500,000	11%	540,881	36%
Mali	2,100,000	420,000	67,200	26,200,000	9%	3,350,592	13%
Mozambique	603,000	120,600	19,296	21,100,000	13%	962,099	5%
Myanmar	2,700,000	540,000	86,400	130,000,000	6%	4,307,904	3%
South Sudan	3,200,000	640,000	102,400	116,400,000	10%	5,105,664	4%
Sudan	4,900,000	980,000	156,800	178,600,000	10%	7,818,048	4%
Ukraine	3,800,000	760,000	121,600	145,000,000	19%	6,062,976	4%
Yemen	9,000,000	1,800,000	288,000	249,400,000	16%	14,359,680	6%
**Total – 19 countries**	62,508,100	12,501,620	2,000,259	**1,935,300,000**		**99,732,924**	
**Average – 19 countries**					12%		5%

Source: data on people targeted (health services in general), requirements for health and funding coverage from Humanitarian Action data for 2024 (Humanitarian Action, 2023a). Est. number of persons in need of mental health services calculated by authors as 20% of people targeted for health services in general, based on WHO data (World health Organization, 2023). Estimated number of adolescents in need of METRA is based on UNICEF estimates that adolescents make up 16% of the population (UNICEF, 2024). Estimated cost of METRA calculated by authors as est. number of persons in need of mental health services at $49.86/person, based on study findings. METRA costs as % total health requirements calculated by authors using table data. METRA costs as percentage of available funding for health are estimated at 39% across all 19 countries, calculated based on estimated cost of METRA as percentage of available funding for health, based on funding coverage (Humanitarian Action, 2023b).
